# High and Low Molecular Weight Hyaluronic Acid Differentially Regulate Human Fibrocyte Differentiation

**DOI:** 10.1371/journal.pone.0026078

**Published:** 2011-10-11

**Authors:** Anu S. Maharjan, Darrell Pilling, Richard H. Gomer

**Affiliations:** 1 Department of Biochemistry and Cell Biology, MS-140, Rice University, Houston, Texas, United States of America; 2 Department of Biology, MS-3474, Texas A&M University, College Station, Texas, United States of America; University Medical Center Freiburg, Germany

## Abstract

**Background:**

Following tissue injury, monocytes can enter the tissue and differentiate into fibroblast-like cells called fibrocytes, but little is known about what regulates this differentiation. Extracellular matrix contains high molecular weight hyaluronic acid (HMWHA; ∼2×10^6^ Da). During injury, HMWHA breaks down to low molecular weight hyaluronic acid (LMWHA; ∼0.8–8×10^5^ Da).

**Methods and Findings:**

In this report, we show that HMWHA potentiates the differentiation of human monocytes into fibrocytes, while LMWHA inhibits fibrocyte differentiation. Digestion of HMWHA with hyaluronidase produces small hyaluronic acid fragments, and these fragments inhibit fibrocyte differentiation. Monocytes internalize HMWHA and LMWHA equally well, suggesting that the opposing effects on fibrocyte differentiation are not due to differential internalization of HMWHA or LMWHA. Adding HMWHA to PBMC does not appear to affect the levels of the hyaluronic acid receptor CD44, whereas adding LMWHA decreases CD44 levels. The addition of anti-CD44 antibodies potentiates fibrocyte differentiation, suggesting that CD44 mediates at least some of the effect of hyaluronic acid on fibrocyte differentiation. The fibrocyte differentiation-inhibiting factor serum amyloid P (SAP) inhibits HMWHA-induced fibrocyte differentiation and potentiates LMWHA-induced inhibition. Conversely, LMWHA inhibits the ability of HMWHA, interleukin-4 (IL-4), or interleukin-13 (IL-13) to promote fibrocyte differentiation.

**Conclusions:**

We hypothesize that hyaluronic acid signals at least in part through CD44 to regulate fibrocyte differentiation, with a dominance hierarchy of SAP>LMWHA≥HMWHA>IL-4 or IL-13.

## Introduction

After tissue injury, local fibroblasts proliferate to repair the wound [1], [2]. In addition to fibroblasts, bone-marrow-derived progenitor cells infiltrate the injured site and differentiate into fibroblast-like cells called fibrocytes [3]. Fibrocytes can differentiate from purified CD14^+^ peripheral blood monocytes, but fibrocytes lose expression of CD14 [4]–[8]. Other studies also suggest that fibrocytes differentiate from a population of bone-marrow derived CD45^+^ CXCR4^+^ cells found in peripheral blood [9]–[11]. Fibrocytes are spindle-shaped cells that express hematopoietic cell markers such as MHC class II, CD34, CD45RO, 25F9, and S100A8/A9, stromal cell markers such as collagen I, and collagen III, and chemokine receptors such as CCR2, CXCR4, and CCR7 that mediate their entry into the site of injury [3], [9], [12]–[15]. Fibrocytes produce cytokines, collagens, angiogenic and fibrogenic growth factors, and matrix metalloproteinases that help to rebuild tissue after injury [3]–[5], [16]–[20]. Fibrocytes are found as a circulating population of cells present in the peripheral blood, and there are elevated numbers of fibrocytes in patients with inflammatory and fibrotic diseases [10], [21], [22]. Peripheral blood monocytes generally become macrophages, and much remains to be understood about the factors that determine whether or not a monocyte becomes a fibrocyte [23].

During tissue injury, the extracellular matrix component hyaluronic acid (HA) breaks down into smaller fragments [24]–[26]. HA is a negatively charged linear polymer of repeating units of (β,1–4)-D-glucuronic acid-(β,1–3)-N-acetyl-D-glucosamine that gives mechanical strength to tissues [27]. High molecular weight hyaluronic acid (HMWHA) has a molecular mass >1×10^6^ Da and is found in normal healthy tissue [28]. The concentration of hyaluronic acid is 15–150 µg/g in lung tissue, 200 µg/g in the vitreous humor of the eye, 500 µg/g in skin, and 1400–3600 µg/g in synovial fluid [29]. In injured tissue, HMWHA breaks down to low molecular weight HA (LMWHA) [24]. LMWHA masses range from 0.8 to 8×10^5^ Da [24]. However, there are variations in the use of the terms HMWHA or LMWHA. HMWHA often refers to any hyaluronic acid that has not been degraded [30], therefore, in this report, we will use HMWHA for hyaluronic acid that is greater than 1×10^6^ Da, LMWHA for 0.8 to 8×10^5^ Da hyaluronic acid, and oligo-HA for <6×10^3^ Da hyaluronic acid. Cells appear to be able to sense the difference between HMWHA, LMWHA, and oligo-HA [25], [31]–[37]. For instance, LMWHA but not HMWHA stimulates alveolar macrophages to secrete inflammatory cytokines such as IL-8 [25], while the maturation and activation of monocyte-derived dendritic cells is promoted by 1.2×10^3^ Da HA, but not HMWHA or LMWHA [36].

One of the major receptors that monocytes and lymphocytes express to detect HA is CD44 [24], [28], [38], [39]. During lung injury, CD44 is used to clear degraded HA [25], [28], [40]. HA-CD44 interactions help the movement of migratory cells during development and help the migration of immune cells into injured sites [24], [27], [41]. HA-CD44 interactions also promote the adhesion and motility of fibroblasts, thus facilitating tissue repair and remodeling of the injured sites [42]. Monocytes, dendritic cells, and lymphocytes also bind HA using Toll-like receptors (TLR) such as TLR2 and TLR4 [32], [33]. LMWHA binds to either TLR2 or TLR4 to elicit pro-inflammatory action, while HMWHA dampens inflammation by inhibiting TLR2 or TLR4 signaling [33], [43]. HA can also bind CD168 (receptor for hyaluronan-mediated motility, RHAMM), a cell-surface receptor on fibroblasts and macrophages [44]. RHAMM is upregulated during inflammation and cancer [44], [45]. Finally, HA can also bind lymphatic vessel endothelial HA receptor (LYVE), which is found predominately in lymphatic endothelial cells and appears to clear HA from lymph [46].

There are several known factors that either promote or inhibit fibrocyte differentiation [6]–[8], [47]–[49]. The pro-fibrotic cytokines IL-4 and IL-13 potentiate the ability of monocytes to differentiate into fibrocytes, while the plasma protein serum amyloid P (SAP), crosslinked IgG, TLR2 agonists, and the pro-inflammatory cytokines IFN-α, IFN-γ and IL-12 inhibit the differentiation of monocytes to fibrocytes [6]–[8], [47], [49]. Since LMWHA increases during tissue injury [24], in this report we examined the effect of hyaluronic acid on fibrocyte differentiation. We found that HMWHA promotes fibrocyte differentiation, while LMWHA inhibits fibrocyte differentiation.

## Materials and Methods

### Culturing PBMC with hyaluronic acid, cytokines, SAP, and antibodies

Blood was collected from healthy adult volunteers with specific approval from the Institutional Review Boards of Rice University and Texas A&M University. Written consent was received and all samples were de-identified before analysis. Peripheral blood mononuclear cells (PBMC) were isolated and incubated in RPMI-based serum free media (SFM) as described previously [7]. High molecular weight hyaluronic acid from rooster comb (HMWHA) and low molecular weight hyaluronic acid from umbilical cord (LMWHA) (both from Sigma-Aldrich, St. Louis, MO) were reconstituted to 25 mg/ml in water. 1,230 Da oligo-HA (Hyalose, Oklahoma City, OK) was reconstituted to 10 mg/ml in water. All experiments were performed with at least three different batches of hyaluronic acid. In some experiments, human serum amyloid P (SAP) (EMD Biosciences, San Diego, CA), recombinant human IL-4 (Peprotech, Rockhill, NJ), or recombinant human IL-13 (Peprotech) was also added to the cells. Before use, SAP was buffer-exchanged as described previously [8]. To ligate surface receptors, PBMC were cultured in SFM with 2 µg/ml anti-human CD44 (clone G44-26) (BD Biosciences, San Jose, CA), anti-human CD44 (clone 515, an antibody that prevents HA binding to CD44) [41], [50] (BD Biosciences), anti-human CD43 (BD Biosciences), or mouse IgG1 isotype control (BD Biosciences). Dilutions of hyaluronic acid (or an equivalent volume of water as a control) were made in SFM. Cells were cultured in duplicate wells of a 96 well tissue culture plate (BD Biosciences). On day 5, fields of PBMC were photographed using a phase-contrast microscope with a 20× objective and viable cells counted as described previously [7]. Cells were then fixed, stained, and the number of fibrocytes was counted, as described previously [6]–[8], [15], [47]. Fibrocytes were identified as adherent spindle-shaped cells with an oval nucleus as described previously [6], [7], [15], [47].

### Treating hyaluronic acid with hyaluronidase

3 mg/ml of HMWHA or 3 mg/ml of LMWHA in PBS were treated with 10 U/ml of hyaluronidase from *Streptomyces hyalurolyticus* (Sigma-Aldrich) for 1 or 4 hours at 37°C, and the digestion was stopped by boiling the solution for 5 minutes at 100°C. Electrophoresis of HA on agarose gels was done following [51] with the exception that 10 µl of sample was mixed with 5 µl of 6× DNA loading buffer [52], and 40 µl of 1 Kb DNA ladder (G5711, Promega, Madison, WI) was mixed with 10 µl of 6× DNA loading buffer [52]. 15 µl of samples or DNA ladder were loaded on the gel, and this was run at 40 V for 8–10 hours [51]. The gels were stained with 0.005% Stains-All (Sigma-Aldrich) in 50% ethanol/water [51]. Dilutions of the digested hyaluronic acids (or PBS as a control) were made in SFM.

### Detecting DNA, RNA, protein, or endotoxin contamination in HMWHA, LMWHA, or digested HMWHA

1 µg/ml 1 Kb DNA ladder, 10 µg/ml, 1 µg/ml, or 0.1 µg/ml of HMWHA, LMWHA, or digested HMWHA were electrophoresed on 1% agarose gels containing 2 µg/ml ethidium bromide for 2 hours at 40 V. The gels were visualized by UV and photographed. We also examined 10 ng/ml, 100 ng/ml, or 1000 ng/ml of HMWHA, or digested HMWHA at 260/280 nm on a Synergy MX (Biotek, Winooski, VT) microplate reader using a Take3 UV plate to examine the presence of DNA, RNA, or protein contamination, using BSA as a protein control. The detection limit of the Synergy MX is 2 ng/µl dsDNA, 2 ng/µl RNA, or 6 ng/µl protein. We tested for the presence of endotoxin in digested HMWHA using THP-1 blue cells (Invivogen, San Diego, CA) and QuantiBlue (Invivogen) following the manufacturer's instructions.

### Preparation of monocytes

CD16-negative monocytes were purified from 5×10^7^ PBMC using an EasySep Monocyte Depletion Kit (StemCell Technology, Vancouver, Canada) following the manufacturer's instructions [7]. To determine the purity of the monocytes, cells were analyzed by flow cytometry (FACScan, BD Biosciences, or Accuri C6 flow cytometer, Ann Arbor, MI), as described previously [7], [8], [15], [53]. A sample of each monocyte preparation was stained with 5 µg/ml primary antibodies against CD3, CD14, CD16, CD19, and CD45, and then incubated with FITC-conjugated F(ab′)2 goat anti-mouse IgG antibodies (cross-adsorbed against human Ig, Southern Biotechnology, Birmingham, AL, USA) as described previously [7], [8]. Only negatively selected cells in excess of 98% pure were used, as determined by the positive expression of CD14 and CD45. Less than 1% of the cells showed staining for the T cell marker CD3, the NK cell marker CD16, or the B cell marker CD19. 100 µl of purified monocytes at 5×10^5^ cells/ml in SFM was mixed with 100 µl of 600 µg/ml HMWHA in SFM, 600 µg/ml LMWHA in SFM, or a water dilution control. On day 5, the cells were fixed, stained, and the number of fibrocytes was counted.

### Immunocytochemistry for fibrocytes

PBMC were cultured on eight-well glass slides (Lab-Tek, Nalge Nunc International, Naperville, IL) in the presence or absence of 300 µg/ml HMWHA or SFM for 5 days, as described previously [53]. Slides were then gently tilted to reduce dislodging cells and the media was then removed from the corner of the well. Immunocytochemistry was performed as described previously [53] with antibodies against CD13 (BioLegend, San Diego, CA), CD14 (BioLegend), CD34 (BD Biosciences), CD45RO (BioLegend), CXCR4 (R&D Systems, Minneapolis, MN), and collagen I (Rockland Immunochemicals, Gilbertsville, PA).

### Immunofluorescence and staining for hyaluronic acid

PBMC were cultured on eight-well glass slides (Lab-Tek, Nalge Nunc International, Naperville, IL) for 5 days, as described previously [8], [53]. Slides were then gently tilted to reduce dislodging cells and the media was then removed from the corner of the well. 400 µl of TBS was then gently added to the wells and then gently pipetted out from the corner of the well. The cells were then fixed with 200 µl of 2% paraformaldehyde (PFA) in PBS for 15 minutes at room temperature. After the PFA was removed, 400 µl of ice-cold methanol was added to the wells for 1 hour at 4°C to permeabilize the cells. After gently removing the methanol, 400 µl of TBS was added to the wells for 10 minutes at room temperature and then gently pipetted out from the corner of the well. This was repeated twice. 400 µl of TBS containing 5% BSA (TBS-5% BSA) was then added to the wells at room temperature for 60 minutes to reduce nonspecific binding. These PBMC slides were then incubated with 5 µg/ml mouse anti-human-CD44 (G44-26), rabbit anti-human-RHAMM (clone EPR4055 Epitomics, Burlingame, CA), or mouse IgG1, isotype control, at room temperature for 60 minutes. Wells were washed with 400 µl of TBS, and then incubated with 2.5 µg/ml FITC-conjugated F(ab′)2 goat anti-mouse or goat anti-rabbit IgG antibodies (Southern Biotechnology, Birmingham, AL) at room temperature for 30 minutes. After washing the slides with TBS, the slides were mounted with Vectashield mounting media containing DAPI (Vector Laboratories, Burlingame, CA). Images of the cells were captured on an Axioplan2 microscope (Zeiss) with a CoolSNAP HQ digital camera (Photometrics, Tucson, AZ) and Metamorph software (Molecular Devices, Dowingtown, PA).

To stain for hyaluronic acid, purified human monocytes were cultured in the presence or absence of 300 µg/ml HMWHA or LMWHA in eight-well glass slides as above for 30 minutes. The media was gently pipetted out and cells were fixed, permeabilized, and non-specific binding reduced as above. The monocytes were then incubated with biotinylated hyaluronic acid binding protein (bio-HABP) (Northstar Associates, East Falmouth, MA) diluted 1∶500 in TBS-5% BSA at room temperature for 60 minutes. Wells were washed with 400 µl of TBS, and then incubated with 1.0 µg/ml streptavidin-FITC (BD Biosciences) at room temperature for 30 minutes. After washing the slides with TBS, the slides were mounted as above.

### Statistics

Statistical analysis was performed using Prism (GraphPad Software, San Diego, CA). Statistical significance was defined as p<0.05 as determined by the statistical methods indicated in the figure legends.

## Results

### HMWHA potentiates fibrocyte differentiation whereas LMWHA inhibits fibrocyte differentiation

**Figure 1:**
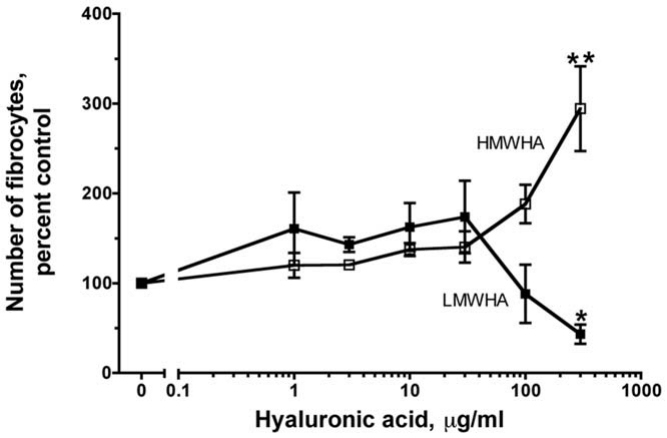
High molecular weight hyaluronic acid (HMWHA) promotes fibrocyte differentiation, while low molecular weight hyaluronic acid (LMWHA) inhibits fibrocyte differentiation. Human PBMC were cultured in the indicated concentrations of HMWHA and LMWHA. After 5 days, the cells were air dried, fixed, stained, and the number of fibrocytes was counted. Fibrocytes were identified as adherent spindle-shaped cells with an oval nucleus. For each donor, values were calculated as the percent of the no-HA control. The results are mean ± SEM (n = 6 separate experiments for HMWHA and n = 5 separate experiments for LMWHA). The number of fibrocytes per 2.5×10^5^ PBMC for the no-HA controls from the 6 donors was 808, 400, 296, 450, 850, and 1200. * indicates p<0.05 and ** p<0.01, compared to control as determined by t-test. Using the non-parametric Mann Whitney two-tailed t-test, 300 µg/ml and 100 µg/ml HMWHA significantly increases the number of fibrocytes when compared to control with p<0.001, and 300 µg/ml LMWHA significantly decreases the number of fibrocytes when compared to control with p<0.01.

**Figure 2:**
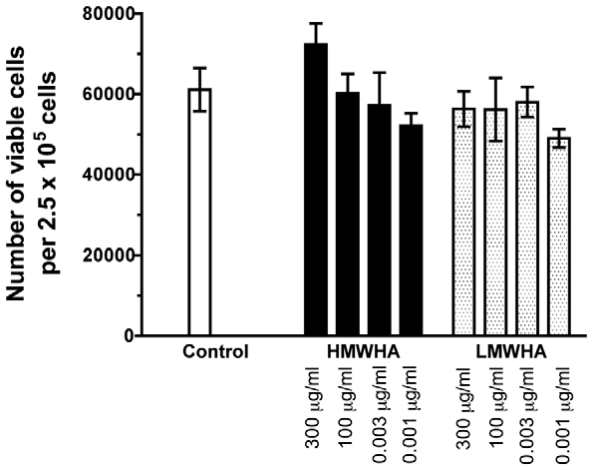
HMWHA and LMWHA do not affect cell viability. Human PBMC were cultured in the absence (SFM) or presence of the indicated concentrations of HMWHA or LMWHA for 5 days. After 5 days, the number of cells was counted. The results are mean ± SEM of viable cells per 0.15 mm^2^ (n = 4 separate experiments). There was no statistical significance as analyzed by ANOVA or t-test.

Hyaluronic acid is a negatively charged glycosaminoglycan that is abundantly present in extracellular matrix [27], [29], [33], [54], [55]. To investigate the role of hyaluronic acid on fibrocyte differentiation, PBMC were cultured in the presence of two different sizes of hyaluronic acid. According to the manufacturer, HMWHA has a range of molecular masses with a peak at 2×10^6^ Da and LMWHA has a range of molecular masses with a peak at 7.5×10^5^ Da; as described below, these sizes were verified by gel electrophoresis. In our serum-free culture media, when PBMC were incubated with HMWHA or LMWHA, we found that 300 µg/ml HMWHA potentiated fibrocyte differentiation, whereas 300 µg/ml LMWHA inhibited fibrocyte differentiation (Figure 1). These data suggest that HMWHA and LMWHA have opposite effects on fibrocyte differentiation. All batches of HMWHA and LMWHA were tested for protein, RNA, DNA, and these were found to be below the level of detection (data not shown). PBMC treated with 300 µg/ml, 100 µg/ml, 0.03 µg/ml, or 0.01 µg/ml HMWHA or LMWHA had no significant difference in the number of viable cells at 5 days compared to untreated cells (Figure 2). These data suggest that HMWHA and LMWHA have opposite effects on fibrocyte differentiation without affecting total cell viability.

To confirm that the long spindle-shaped cells are fibrocytes, we stained day 5 PBMC incubated with 300 µg/ml HMWHA or SFM for fibrocyte markers such as CD13, CD34, CD45RO, CXCR4, and collagen I. When PBMC were cultured in the presence of 300 µg/ml HMWHA or SFM for 5 days, the long spindle-shaped cells stained positively for CD13, CD34, CD45RO, CXCR4, and collagen I (data not shown). We also stained the day 5 PBMC cultured in 300 µg/ml HMWHA or SFM for the monocyte marker CD14 and the macrophage marker PM2K. Fibrocytes from both conditions did not stain for CD14 or PM2K (data not shown). There were few non-fibrocyte cells that stained positively for CD14 and PM2K (data not shown). Together, these observations indicate that the long spindle-shaped cells are fibrocytes.

### Hyaluronidase treated HA inhibits fibrocyte differentiation

**Figure 3:**
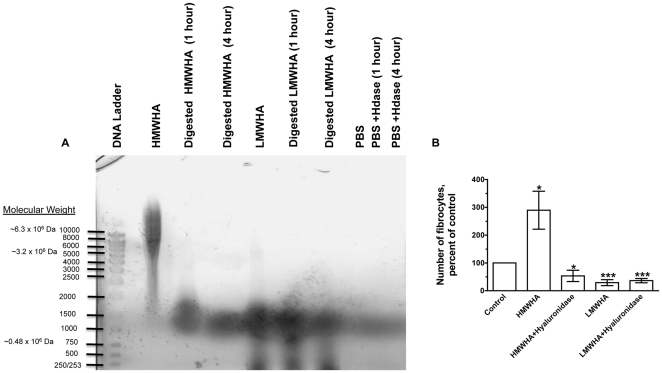
HMWHA and LMWHA treated with hyaluronidase inhibit fibrocyte differentiation. (**A**) HMWHA and LMWHA were treated with 10 U/ml of hyaluronidase for 1 and 4 hours at 37°C. Samples were analyzed by agarose gel electrophoresis. M, 1 Kb ladder; Lane 1, HMWHA; Lane 2, HMWHA incubated with hyaluronidase for 1 hour; Lane 3, HMWHA incubated with hyaluronidase for 4 hours; Lane 4, LMWHA; Lane 5, LMWHA incubated with hyaluronidase for 1 hour; Lane 6, LMWHA incubated with hyaluronidase for 4 hours; Lane 7, PBS; Lane 8, PBS incubated with hyaluronidase for 1 hour; and Lane 9, PBS incubated with hyaluronidase for 4 hours. (**B**) Human PBMC were cultured in 300 µg/ml of 4 hours-hyaluronidase-treated or untreated HMWHA and LMWHA. After 5 days, the cells were air dried, fixed, stained, and the number of fibrocytes was counted. The results are mean ± SEM of SFM percent control (n = 6 separate experiments). The number of fibrocytes in SFM controls from the 6 donors was 883, 1029, 808, 288, 400, and 288. * indicates p<0.05, ** p<0.01, *** p<0.001 as determined by t-test. Using the non-parametric Mann Whitney two-tailed t-test, 300 µg/ml HMWHA significantly increases the number of fibrocytes when compared to control with p<0.001, but 300 µg/ml hyaluronidase incubated HMWHA is not significantly different from control. Also, 300 µg/ml LMWHA significantly decreases the number of fibrocytes when compared to control with p<0.001, and 300 µg/ml hyaluronidase incubated LMWHA also significantly decreases the number of fibrocytes when compared to control with p<0.001. Using the non-parametric Mann Whitney one-tailed t-test, 300 µg/ml hyaluronidase incubated HMWHA significantly decreases the number of fibrocytes when compared to control with p<0.05.

Since HMWHA potentiated fibrocyte differentiation and LMWHA inhibited fibrocyte differentiation, it is possible that HMWHA contains a contaminant that potentiates fibrocyte differentiation, or LMWHA contains a contaminant that inhibits fibrocyte differentiation. To confirm that hyaluronic acid of different sizes have different effects, we digested HMWHA into smaller fragments with hyaluronidase to determine if digested HMWHA inhibits fibrocyte differentiation. HMWHA had a range of molecular mass of approximately 6.3×10^6^ Da–2×10^6^ Da (Figure 3A). The size range of the HMWHA digested with hyaluronidase was equivalent to the size of LMWHA (a mass range of approximately 6×10^5^ Da–8×10^5^ Da) as analyzed by gel electrophoresis, while hyaluronidase treatment of LMWHA slightly decreased the upper end of the size range of the LMWHA (Figure 3A). Hyaluronidase may have digested the HMWHA and LMWHA into smaller sizes such as oligo hyaluronic acid. However, we were unable to detect these smaller sizes on the gel. Hyaluronidase-treated HMWHA and LMWHA inhibited fibrocyte differentiation (Figure 3B). Hyaluronidase-containing PBS had no effect on fibrocyte differentiation (data not shown). Since HMWHA were digested with bacterial hyaluronidase, it is possible that the digested HMWHA may contain endotoxins that are actually causing the inhibition of fibrocyte differentiation. Hyaluronidase digested HMWHA always had endotoxin below detectable levels (data not shown). Treatment of PBMC with HMWHA and LMWHA digested with hyaluronidase also did not affect cell viability at 5 days (data not shown). Together, this suggests that the potentiation of fibrocyte differentiation by HMWHA and the inhibition of differentiation by LMWHA are not due to a non-HA contaminant.

**Figure 4:**
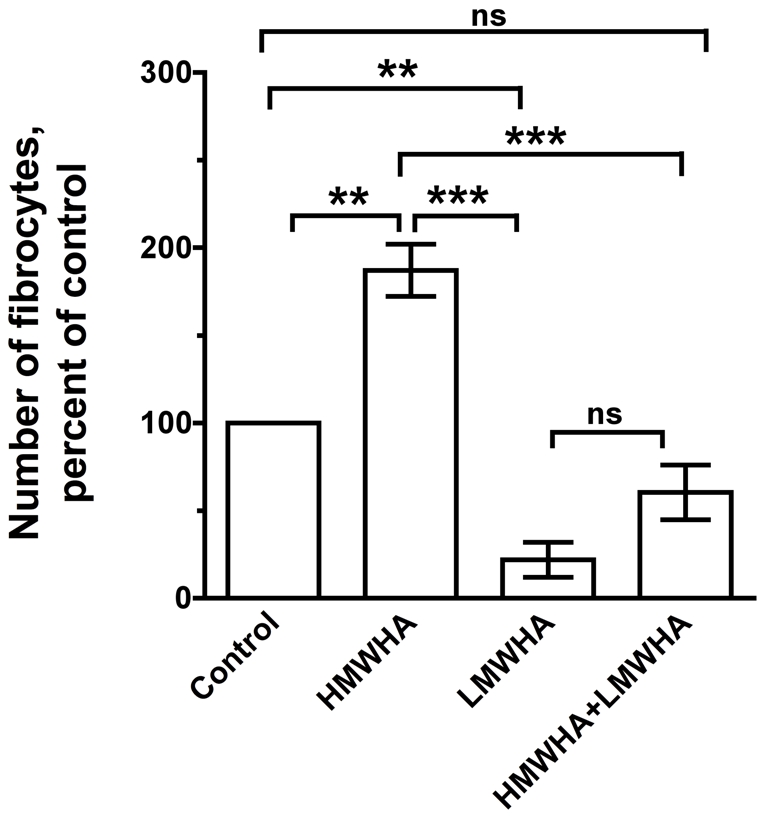
LMWHA inhibits HMWHA-induced fibrocyte differentiation. Human PBMC were cultured in SFM (control), SFM with 300 µg/ml HMWHA, SFM with 300 µg/ml LMWHA, or SFM with 300 µg/ml HMWHA and 300 µg/ml LMWHA. After 5 days, the cells were air dried, fixed, stained, and the number of fibrocytes was counted. The results are mean ± SEM of percent of control (n = 3 separate experiments). The number of fibrocytes per 2.5×10^5^ PBMC in controls from the 3 donors was 1525, 713, and 1290. * indicates p<0.05, ** p<0.01, *** p<0.001 compared to control as determined by ANOVA. Using the non-parametric Mann Whitney two-tailed t-test, there is no statistical significant difference among different experimental groups. Using the non-parametric Mann Whitney one-tailed t-test, 300 µg/ml HMWHA significantly increases the number of fibrocytes when compared to control with p<0.05, 300 µg/ml LMWHA significantly decreases the number of fibrocytes when compared to control with p<0.05, 300 µg/ml HMWHA and 300 µg/ml LMWHA significantly decreases the number of fibrocytes when compared to control with p<0.05. Additionally, 300 µg/ml HMWHA significantly increases the number of fibrocytes when compared to 300 µg/ml LMWHA or the combination of 300 µg/ml HMWHA and LMWHA with p<0.05. 300 µg/ml LMWHA significantly decreases the number of fibrocytes when compared to 300 µg/ml HMWHA or the combination of 300 µg/ml HMWHA and LMWHA with p<0.05.

High and low molecular weight HA are present at sites of injury [24], [34], [56]. We therefore examined how combinations of 300 µg/ml HMWHA and 300 µg/ml LMWHA affect fibrocyte differentiation. The number of fibrocytes observed in PBMC treated with a combination of HMWHA and LMWHA was significantly lower compared to PBMC cultured with HMWHA alone, but not significantly different compared to PBMC cultured with LMWHA alone (Figure 4). In this experiment, we added 2.7 times more moles of LMWHA than HMWHA. It is possible that the increased amount of LMWHA that we added neutralized the effect of HMWHA on fibrocyte differentiation. When we analyze the data with Mann Whitney one-tailed t-tests, we observe that 300 µg/ml HMWHA or 300 µg/ml LMWHA are significantly different from the combination of 300 µg/ml HMWHA and LMWHA, which suggests that HMWHA and LMWHA both neutralize each other's effect on fibrocyte differentiation.

### HMWHA and LMWHA directly affect the differentiation of monocytes to fibrocytes

**Figure 5:**
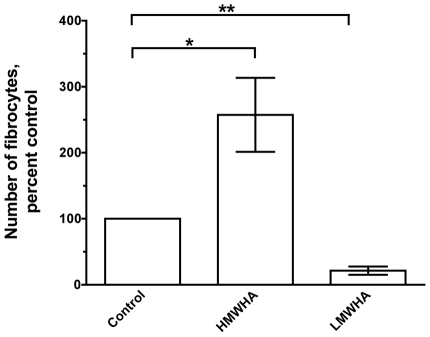
HMWHA potentiates but LMWHA inhibits the differentiation of purified monocytes to fibrocytes. Purified human monocytes were cultured in 300 µg/ml of either HMWHA or LMWHA for 5 days. After 5 days, the cells were air dried, fixed, stained, and the number of fibrocytes was counted. The results are mean ± SEM of percent no-HA control (n = 3 separate experiments). The number of fibrocytes per 2.5×10^5^ monocytes in controls from the 3 donors was 4250, 2450, and 1600. * indicates p<0.05 and ** indicates p<0.01 compared to control as determined by t-test. Using the non-parametric Mann Whitney two-tailed t-test, there is no significant difference among the control, 300 µg/ml HMWHA, or 300 µg/ml LMWHA. Using the non-parametric Mann Whitney one-tailed t-test, 300 µg/ml HMWHA significantly increases the number of fibrocytes when compared to control, 300 µg/ml LMHWA significantly decreases the number of fibrocytes when compared to control.

We previously found that IL-12 and TLR2 agonists, when added to a population of PBMC, indirectly affect monocyte to fibrocyte differentiation [7], [8], while factors such as SAP, IL-4, IL-13, or aggregated IgG directly affect monocyte to fibrocyte differentiation [6], [8], [47]. Therefore, we examined whether HMWHA or LMWHA act directly on human monocytes. When 300 µg/ml HMWHA or LMWHA were added to purified monocytes, HMWHA potentiated fibrocyte differentiation while LMWHA inhibited fibrocyte differentiation (Figure 5). This suggests that HMWHA and LMWHA act directly on monocytes to either potentiate or inhibit fibrocyte differentiation.

### Oligo hyaluronic acid has no effect on fibrocyte differentiation

**Figure 6:**
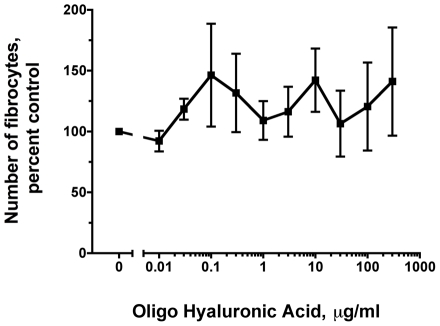
Oligo hyaluronic acid does not affect fibrocyte differentiation. Human PBMC were cultured in different concentrations of oligo hyaluronic acid for 5 days. After 5 days, the cells were air dried, fixed, stained, and the number of fibrocytes was counted. The results are mean ± SEM of percent control (n = 3 separate experiments). The number of fibrocytes 2.5×10^5^ PBMC in controls from the 3 donors was 4150, 2560, and 575.

Since LMWHA and hyaluronidase-treated HMWHA decreased the number of fibrocytes, we investigated the possibility that very small hyaluronic acid polymers might inhibit fibrocyte differentiation. When PBMC were cultured with increasing concentrations of 6-mer oligo hyaluronic acid (1,230 Da), there was no difference in the number of fibrocytes compared to SFM (Figure 6). This indicates that although LMWHA inhibits fibrocyte differentiation, the effect requires a polymer larger than a 6-mer/1,230 Da hyaluronic acid. This also suggests that hyaluronidase digested HMWHA inhibited fibrocyte differentiation (Figure 3B) due to a size that is similar to LMWHA, and not because of the small fragment sizes that are similar to oligo hyaluronic acid.

### HMWHA and LMWHA have different effects on the expression of CD44

**Figure 7:**
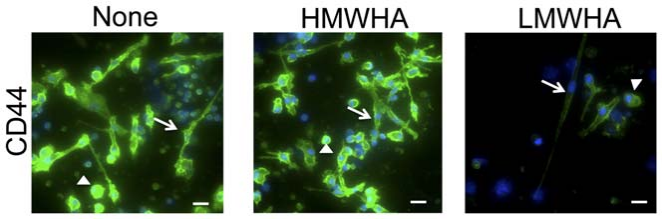
HMWHA and LMWHA have different effects on the expression of CD44. PBMC were incubated in SFM, SFM with 300 µg/ml HMWHA, or SFM with 300 µg/ml LMWHA for 5 days. The PBMC were then fixed and stained for CD44. In all panels, nuclei are stained blue with DAPI and bars are 20 µm. Arrows indicate fibrocytes and arrowheads indicate non-fibrocyte cells. The figures represent one of 3 independent experiments.

Hyaluronic acid binds to the cell-surface molecule CD44, and this interaction plays an important role in development, inflammation, tumor growth, and the recruitment and activation of many immune cells into injured tissues [57]. Therefore we tested the effect of HMWHA and LMWHA on the expression of CD44 on PBMC. After culturing PBMC in the presence or absence of 300 µg/ml of HMWHA or 300 µg/ml of LMWHA for 5 days, cells were stained for CD44. For PBMC cultured in SFM, both fibrocytes and non-fibrocyte cells expressed CD44 (Figure 7). In cultures containing HMWHA, fibrocytes and non-fibrocyte cells showed staining for CD44 similar to the control (Figure 7). In cultures containing LMWHA, both fibrocytes and non-fibrocyte cells had less staining for CD44 compared to the control (Figure 7). These results indicate that not only do HMWHA and LMWHA have opposite effects on fibrocyte differentiation, but they also have different effects on the levels of CD44 on fibrocytes and non-fibrocyte cells.

### Anti-human CD44 antibodies potentiate fibrocyte differentiation

**Figure 8:**
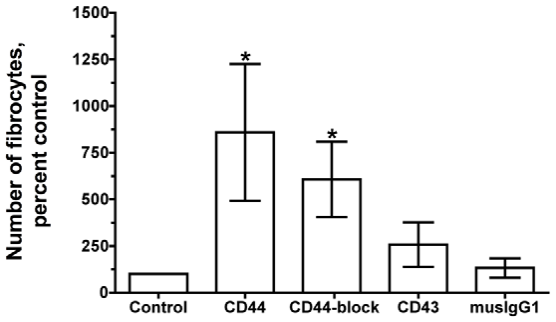
Anti-CD44 antibodies potentiate the differentiation of monocytes to fibrocytes. Human PBMC were cultured in 2 µg/ml anti-human anti-CD44, anti-CD43, or mouse IgG1 isotype control. After 5 days, the cells were air dried, fixed, stained, and the number of fibrocytes was counted. The results are mean ± SEM of percent control (n = 6 separate experiments). The number of fibrocytes per 2.5×10^5^ PBMC in controls was 350, 50, 350, 250, 450, and 50. * indicates p<0.05 compared to control, as determined by ANOVA with Dunnett's post-test). Using the Mann Whitney two-tailed t-test, 2 µg/ml anti-human anti-CD44 antibodies significantly increases the number of fibrocytes when compared to control with p<0.001.

To determine whether the hyaluronic acid binding receptor CD44 could directly affect fibrocyte differentiation, we cultured PBMC with two different anti-human CD44 antibodies, anti-human CD43, or mouse IgG1 isotype control for 5 days. We used CD43 as a second hematopoietic marker as it plays an important role in adhesion, cell-signaling, and cytoskeleton interactions, but does not bind HA [58]. One of the anti-human CD44 antibodies (clone 515) is a blocking antibody that binds at or near the same site as HA on CD44, preventing HA from binding to CD44 [41], [50]. The second CD44 mAb (clone G44-26) does not prevent HA binding to CD44. When added to PBMC, both CD44 antibodies significantly increased the number of fibrocytes compared to PBMC cultured in either SFM or mouse IgG1 antibodies (Figure 8). Anti-human CD43 and mouse IgG1 isotype control antibodies had no statistically significant effect on fibrocyte differentiation.

HA can also bind to other surface receptors such as RHAMM [59], [60]. We examined if HMWHA or LMWHA affect the expression of RHAMM. After culturing PBMC in the presence or absence of 300 µg/ml of HMWHA or 300 µg/ml of LMWHA for 5 days, cells were stained for RHAMM. As described previously in activated monocytes [61] or CD34^+^ cells [62], we did not observe any staining for RHAMM in fibrocytes or non-fibrocyte cells in the presence or absence of HMWHA or LMWHA (data not shown).

### Purified monocytes internalize HMWHA and LMWHA

**Figure 9:**
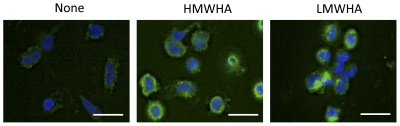
At 30 minutes, both LMWHA and HMWHA are internalized by monocytes. Purified human monocytes were incubated in SFM, SFM with 300 µg/ml LMWHA, or 300 µg/ml of HMWHA for 30 minutes. The PBMC were stained with biotinylated hyaluronic acid binding protein (HABP) and this was detected with streptavidin-FITC. In all panels, nuclei are stained blue with DAPI and bars are 20 µm. The figures represent one of the 3 independent experiments.

As HMWHA and LMWHA directly affect the differentiation of monocytes to fibrocytes, we examined if the internalization of HMWHA and LMWHA was different in purified monocytes. Purified monocytes were incubated with or without 300 µg/ml of HMWHA or 300 µg/ml of LMWHA, and after 30 minutes the cells were fixed. The fixed cells were then stained for hyaluronic acid. We detected a low intensity fluorescence signal on untreated monocytes, which could have been due to autofluorescence, non-specific binding of the biotinylated hyaluronic acid binding protein (bio-HABP), or the presence of HA bound to the cells ex vivo, as these were freshly prepared cells, and HA is present in plasma at 0.01–0.03 µg/ml [63]. We observed HA staining inside HMWHA and LMWHA treated monocytes (Figure 9). Monocytes treated with HMWHA or LMWHA for 60 minutes also had HA staining inside the cells (data not shown). This suggests that the opposite effect of HMWHA and LMWHA on fibrocyte differentiation is unlikely to be due to differential internalization of high and low molecular weight hyaluronic acids by purified monocytes. Although ten-day cultured fibrocytes synthesize hyaluronic acid [64], our inability to detect hyaluronic acid on monocytes cultured for 60 minutes suggest that hyaluronic acid production in fibrocytes is developmentally regulated.

### SAP inhibits HMWHA-induced fibrocyte differentiation and potentiates LMWHA-induced fibrocyte inhibition

**Figure 10:**
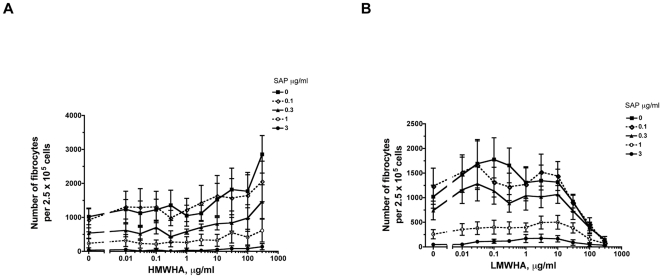
SAP inhibits HMWHA-induced fibrocyte differentiation and potentiates LMWHA-induced fibrocyte inhibition. (**A**) Human PBMC were cultured in different concentrations of HMWHA with 0, 0.1, 0.3, 1, and 3 µg/ml of SAP. After 5 days, the cells were air dried, fixed, stained, and the number of fibrocytes was counted. The results are mean ± SEM of fibrocytes per 2.5×10^5^ PBMC (n = 5 separate experiments, except n = 11 separate experiments for HMWHA with no SAP). With no SAP, the difference between no treatment and 300 µg/ml HMWHA is significant with p<0.01 (t-test). With 300 µg/ml HMWHA, adding 1 or 3 µg/ml SAP significantly decreases the number of fibrocytes with p<0.05 (ANOVA). Using the non-parametric Mann Whitney two-tailed t-test, with no SAP, the difference between no treatment and 300 µg/ml HMWHA is significant with p<0.05. With 300 µg/ml HMWHA, adding 1 or 3 µg/ml SAP significantly decreases the number of fibrocytes with p<0.05. (**B**) Human PBMC were cultured in different concentrations of LMWHA with 0, 0.1, 0.3, 1, and 3 µg/ml of SAP for 5 days. After 5 days, the cells were air dried, fixed, stained, and the number of fibrocytes was counted. The results are mean ± SEM of fibrocytes per 2.5×10^5^ PBMC (n = 5 separate experiments, except n = 11 separate experiments for LMWHA with no SAP). With no added SAP, the difference between no treatment and 100 µg/ml or 300 µg/ml LMWHA is significant with p<0.001 (t-test). Using the non-parametric Mann Whitney two-tailed t-test, with no added SAP, the difference between no treatment and 100 µg/ml or 300 µg/ml LMWHA is significant with p<0.05.

A variety of pro-fibrocyte and anti-fibrocyte factors are present during tissue injury [8], [24], [48], [49], [65]. We investigated the effect of HMWHA and LMWHA on fibrocyte differentiation in the presence of SAP, a potent inhibitor of fibrocyte differentiation [6], [8], [15], [53], [66]–[69]. PBMC were cultured in different concentrations of HMWHA or LMWHA along with different concentrations of SAP. SAP counteracted HMWHA-induced fibrocyte differentiation, and potentiated LMWHA-induced fibrocyte inhibition (Figure 10). This suggests that the fibrocyte-inhibiting activity of SAP has a dominant effect over the fibrocyte-promoting activity of HMWHA, and that LMWHA potentiates the ability of SAP to inhibit fibrocyte differentiation.

### LMWHA inhibits IL-4 or IL-13-induced fibrocyte differentiation

**Figure 11:**
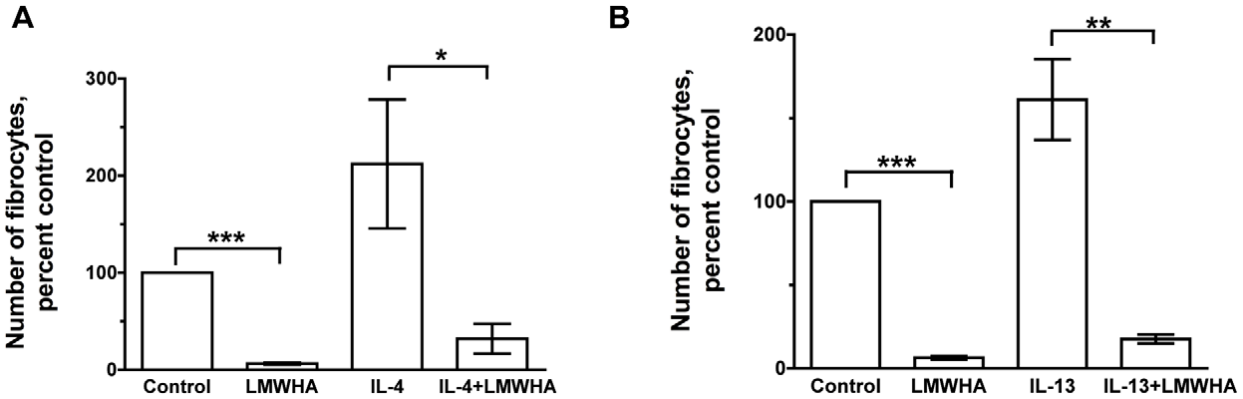
LMWHA inhibits IL-4 or IL-13-induced fibrocyte differentiation. Human PBMC were cultured in (**A**) SFM, SFM with 1 ng/ml IL-4, SFM with 300 µg/ml LMWHA, or SFM with 1 ng/ml IL-4 and 300 µg/ml LMWHA, or (**B**) SFM, SFM with 1 ng/ml IL-13, or SFM with 1 ng/ml IL-13 and 300 µg/ml LMWHA. After 5 days, the cells were air dried, fixed, stained, and the number of fibrocytes was counted. The results are mean ± SEM of percent control (n = 3 separate experiments). The number of fibrocytes per 2.5×10^5^ PBMC in controls from the 3 donors was 900, 1520, and 431. * indicates p<0.05, ** p<0.01, and *** p<0.001 compared to control as determined by t-test. Using the non-parametric Mann Whitney two-tailed t-test, there is no significant difference among control, 300 µg/ml LMWHA, 1 ng/ml IL-4, 1 ng/ml IL-13, the combination of 300 µg/ml LMWHA and 1 ng/ml IL-4, or the combination of 300 µg/ml LMWHA and 1 ng/ml IL-13. Using the non-parametric Mann Whitney one-tailed t-test, 300 µg/ml LMWHA significantly decreases the number of fibrocytes when compared to control with p<0.05. Also, the combination of 300 µg/ml LMWHA and 1 ng/ml IL-4 significantly decreases the number of fibrocytes when compared to 1 ng/ml IL-4 with p<0.05. The combination of 300 µg/ml LMWHA and 1 ng/ml IL-13 also significantly decreases the number of fibrocytes when compared to 1 ng/ml IL-13 with p<0.05.

IL-4 and IL-13 are cytokines that potentiate fibrocyte differentiation [6], [8], [53]. To determine if there is a regulatory hierarchy, we examined the effect of LMWHA on fibrocyte differentiation in the presence of IL-4 or IL-13. IL-4 and IL-13 promoted fibrocyte differentiation, and LMWHA inhibited IL-4- and IL-13-induced fibrocyte differentiation (Figure 11). This suggests that the fibrocyte inhibiting activity of LMWHA has a dominant effect over the fibrocyte promoting activities of IL-4 and IL-13.

## Discussion

We found that 300 µg/ml HMWHA potentiates fibrocyte differentiation and 300 µg/ml LMWHA inhibits fibrocyte differentiation. These HA concentrations are similar to what has been observed in tissues [29], and the HA concentrations used in other studies [25], [28], [33], [70], [71]. The effect of these two different sizes of hyaluronic acid on fibrocyte differentiation is a direct effect on monocytes. HMWHA and LMWHA also have different effects on the levels of the adhesion receptor CD44. The opposite effect of HMWHA and LMWHA on fibrocyte differentiation does not appear to be due to the difference in the internalization of HMWHA and LMWHA by monocytes. Anti-human CD44 antibodies potentiate fibrocyte differentiation, suggesting that CD44 may at least in part mediate the effect of hyaluronic acid on fibrocyte differentiation. SAP inhibits HMWHA-induced fibrocyte differentiation and potentiates LMWHA-induced fibrocyte differentiation, while LMWHA inhibits IL-4- or IL-13-potentiated fibrocyte differentiation. These results suggest a dominance hierarchy of SAP>LMWHA≥HMWHA>IL-4 or IL-13.

HMWHA and LMHWA bind to CD44, TLR2, TLR4, LYVE, and RHAMM (CD168) receptors to accomplish their biological effects [56]. We have previously shown that TLR2 and TLR4 agonists do not have a direct effect on monocyte to fibrocyte differentiation [7]. Therefore, it is unlikely that HWMHA and LMWHA regulate fibrocyte differentiation via TLR2 or TLR4. We and others have also failed to detect RHAMM expression on fibrocytes or human monocytes cultured ex vivo, although macrophage cell lines express RHAMM [61], [62]. Finally LYVE is a HA receptor expressed predominately by lymphatic endothelium [46], and we have not detected LYVE on monocytes, macrophages, or fibrocytes (data not shown) [53]. As antibodies against CD44 potentiated fibrocyte differentiation, CD44 appears to be the dominant receptor for HA induced regulation of fibrocyte differentiation.

Several studies have described differential effects of HMWHA and LMWHA on different types of cells such as macrophages, dendritic cells, osteoclasts, and T cells [25], [32]–[37]. For example, in murine and human macrophages, HMWHA (6×10^6^ Da) did not affect the secretion of pro-inflammatory chemokines or cytokines [25], while LMWHA (<5×10^5^ Da) stimulated these cells to produce inflammatory chemokines such as macrophage inflammatory protein-1α (MIP-1α), macrophage inflammatory protein-1β (MIP-1β), and monocyte chemoattractant protein-1 (MCP-1) [25], [32], [72], [73]. In cultured macrophages, HMWHA (4.6×10^5^–2.8×10^6^ Da) inhibited phagocytosis, while LMWHA (9.0×10^4^ Da) enhanced phagocytosis [71]. The inhibition of macrophage phagocytosis was proportional to the molecular weight of the HA. These results suggest that LMWHA (<5×10^5^ Da) actively stimulate many types of cells at sites of injury to facilitate the clearance of debris and infectious agents, while HMWHA (>10^6^ Da), which is the dominant HA in resolving wounds, acts as a signal for repair.

During tissue injury, cirrhosis, and fibrosis, the concentration of HA increases in serum and tissue fluids, and this HA appears to be mainly LMWHA [24], [33], [55]. The normal range of HA in serum is 0.01–0.1 µg/ml, while in patients with liver injury the serum concentration of HA reaches 0.1–0.3 µg/ml [63]. In murine models of lung injury, the concentration of HA increased by 50% and the size of the accumulated HA was approximately 5.4×10^5^ Da [28]. As the injury resolved, the concentration of HA returned to basal levels and the size reverted to 1.4×10^6^–3.1×10^6^ Da, [28], [74] similar to the size of HA in the lungs of control mice. These results show that following an insult, there is an increase in the concentration of small hyaluronic acid fragments. As the injury resolves, the concentration of HA decreases and the size returns back to normal (>10^6^ Da). Combined with our results, this suggests that the presence of LMWHA at an injured site would inhibit fibrocyte differentiation and collagen deposition to allow macrophages to freely move about the injured site to phagocytose debris and clear any infectious agents. As wound healing progresses and the ratio of HMWHA to LMWHA increases [27], the HMWHA will promote monocyte to fibrocyte differentiation, leading to tissue repair with the fibrocytes secreting ECM and also stimulating ECM production by fibroblasts [19], [20], [48]. However, there is still no known mechanism to explain how a simple repeating disaccharide of varying length has opposing effects on a same type of cell.
